# Teaching approaches and satisfaction of medical students during anesthesia rotations: a multicenter cross-sectional study

**DOI:** 10.1186/s12909-023-04603-8

**Published:** 2023-09-07

**Authors:** Ramzi Shawahna, Mohammad Jaber, Iyad Maqboul, Mansour Khaleel, Jenan Abo-Mokh, Hisham Sabbah, Sudqi Assi, Jehad Zuhd

**Affiliations:** 1 Department of Physiology, Pharmacology and Toxicology, Faculty of Medicine and Health Sciences, An-Najah National University, New Campus, Building: 19, Office: 1340, P.O. Box 7, Nablus, Palestine; 2 Clinical Research Center, An-Najah National University Hospital, 44839 Nablus, Palestine; 3 Department of Medicine, Faculty of Medicine and Health Sciences, An-Najah National University, Nablus, Palestine; 4 An-Najah National University Hospital, An-Najah National University, Nablus, Palestine

**Keywords:** Anesthesiology, Medical education, Medical students, Anesthesia training, Palestine

## Abstract

**Background:**

Anesthesia training is an important component of medical education. This multicenter study was conducted to determine the teaching approaches used during anesthesia training or rotations and to assess the satisfaction of the medical students.

**Methods:**

This multicenter study was conducted in a cross-sectional design. The study was conducted among 5th and 6th year medical students who completed their anesthesia rotations in different training centers in Palestine. The data were collected using a questionnaire in adherence to the strengthening the reporting of observational studies in epidemiology statement.

**Results:**

Questionnaires were returned by 385 medical students. The mean anesthesia rotation length was 12.4 ± 2.1 days. On average, the students witnessed 7.8 ± 4.9 procedures performed under general, regional, and local anesthesia in a week. Of the students, 135 (35.1%) and 126 (32.8%) stated that the educators always or often explained how and why they did procedures or techniques during the anesthesia rotation and assessed their baseline level of knowledge before giving new knowledge or explaining things. On the other hand, stepping back and allowing the trainees to work through, presenting articles or literature relevant to the case, and being open to trying new or different procedures or techniques were less often reported by the medical students. Less than half of the students were satisfied with their competencies gained through the anesthesia rotation.

**Conclusion:**

Educators used active and effective teaching or training approaches less frequently during the anesthesia rotations. The findings of this study also showed that the medical students were not satisfied with their competencies after their anesthesia rotations. More studies are still needed to determine the best ways to improve anesthesia rotations and medical education or training in Palestine.

## Background

Anesthesia is one of the high-risk medical specialties in which the ability to proficiently perform practical procedures is vital to patient safety and the provision of high-quality care services [1, 2]. In clinical practice, anesthesiologists need to proficiently conduct preoperative patient assessments, perform intraoperative anesthesia procedures, and provide postoperative care and pain relief [3]. These competencies require different skills and knowledge that the anesthesiologists need to acquire during their medical education and training.

Anesthesia training is an important component of medical education that medical students often receive in the late stages of their medical education. In different educational systems, the anesthesia rotation is composed of a supervised clinical experience in which the trainees are supposed to gain competencies in the 6 areas: medical knowledge, patient-centered care, practice-based learning and improvement, professionalism, interpersonal and communication skills, and systems-based practice [3, 4]. During this heavily task-oriented experience, the trainees are exposed to different clinical situations and learn how to care for the patients [5]. In Palestine, medical students receive a two-week anesthesia rotation in the 5th year of their medical education. During this supervised clinical-based rotation, the medical students spend their mornings in operating theaters and recovery rooms to learn the principles of anesthesia under the supervision of practicing anesthesiologists. The intended learning outcomes of the anesthesia training include understating the basics of pre-operative assessment of patients, airway and fluid management, types of anesthesia, indications, and contraindications of anesthesia, induction, and maintenance of anesthesia, complications associated with anesthesia, monitoring and recovery of patients, differential diagnosis of hypoxemia and hypercarbia, and indications and practice of mechanical ventilation and resuscitation. In addition to the clinical training, the medical students receive daily seminars that focus on the important aspects of anesthesia including perioperative care, pharmacology of anesthetics, obstetric anesthesia, pediatric anesthesia, pain management, and complications of anesthesia. The medical students are also trained on how to prepare a care plan for patients undergoing general and regional anesthesia, how to deal with difficult intubation procedures, and how to deal with high-risk patients. In addition to the supervised hands-on training, the teaching methods include lectures, seminars, case studies, group discussions, assignments, and preparation of care plans.

Recently, medical education has transitioned from process- and structure-oriented education to competency-based assessment of outcomes [6]. Despite this transition, little guidance is currently available for educators on the best ways to improve the competencies of trainees in anesthesia. It has been argued that adequate training of current and future healthcare professionals is a prerequisite for safe and effective healthcare delivery. Therefore, medical educators should ensure that the courses and training sessions are designed and delivered in effective ways to optimize the competencies of the trainees [3, 6, 7]. However, assessing competencies in hospital-based specialties like anesthesiology has been recognized as problematic [7, 8]. Hausman et al. conducted a study to assess the status of residency programs in training residents in office-based anesthesia [9]. The study concluded that residents were minimally exposed to office-based anesthesia during their training.

Little studies were conducted to evaluate teaching or training approaches in anesthesia. Additionally, few studies were conducted to assess the satisfaction and performance of the trainees after completing anesthesia rotations. Therefore, this study was conducted to evaluate the anesthesia training of medical students in Palestine. The objectives of this study were to determine the teaching and training approaches used by the trainers during the anesthesia rotations and the satisfaction of the trainees.

## Methods

### Study settings and context

This study was conducted among medical students in all medical schools in Palestine. The study was conducted in the context of assessing the anesthesia training of future physicians in Palestine. In Palestine, the Doctor of Medicine (MD) program consists of 130 credit hours of basic courses that are followed by 135 credit hours of clinical training [10]. Medical students can complete their MD degree program in 6 academic years. In Palestine, medical students attend anesthesia rotations in their 5th year of medical education.

### Study design

This multicenter study was conducted in a cross-sectional design. The study was conducted and reported in adherence to the strengthening the reporting of observational studies in epidemiology (STROBE) statement [11]. The statement ensured reporting the study objectives, settings and context, population, sampling, and recruitment of participants, representativeness, study tool, validity and reliability of the study tool, data analysis, the main results of the study, interpretation of the main findings, discussion of the strengths and limitations, and generalizability.

### Population

The target population of the study was medical students in the 5th or 6th year of their medical education or training. This population was defined by the completion of the anesthesia rotations.

### Sample size, recruitment of participants, and sampling

The sample size needed for this study was calculated using an online sample size calculator (www.raosoft.com). The population was the sum of all medical students in the 5th or 6th year of their medical education or training who completed their anesthesia rotations. The total number of medical students who were in the 5th and 6th years of their medical education in all medical schools in Palestine was about 1,500. Accounting for a larger population of 2,000 medical students, the sample size was calculated at a 95% confidence interval (95% CI) accepting a margin of error of 5%. The sample size needed for this study was 323 medical students.

The medical students were recruited by the field researchers using a convenience sampling approach. The following inclusion criteria were used to select the potential participants: (1) medical students in their 5th or 6th year, (2) having completed anesthesia rotations, (3) expressing willingness to participate in the study, and (4) provision of informed consent. The medical students who did not meet the inclusion criteria were excluded from this study.

### The study tool

In this study, the study tool was a questionnaire that was informed by previous studies [8, 9, 12–17]. Additionally, the Doctor of Medicine (MD) clinical handbooks and the formally approved curricula of the anesthesia course/rotation were also searched. The MD clinical handbooks contain information and instructions about the clinical rotations that the medical students have to attend, the timetables, and the rotation maps for the clinical courses. The items were selected based on their suitability and relevance to the intended learning objectives of the anesthesia rotation offered to the medical students in Palestine. The questionnaire consisted of three parts. In the first part, the medical students were asked to provide their demographic, academic, and training variables of the medical students like gender, age, academic year, length of anesthesia rotation, length of surgery rotation, the approximate number of students in the anesthesia rotation, and the approximate number of procedures performed under general, regional, or local anesthesia in a week. Additionally, the medical students were asked to self-rate their satisfaction with their academic achievements, knowledge of anesthesia compared to other clinical subjects, and the influence of the anesthesia rotation on their awareness of anesthesia. Moreover, the students were also asked whether they would consider a future career in anesthesia. In the second part, the medical students were asked to report the frequency of teaching or training approaches used by the educators during their anesthesia rotation using a 5-point Likert scale (1 = never, 5 = always). The approaches included stepping back and allowing the trainees to work through, explaining how and why the educators did procedures or techniques, presenting articles or literature relevant to the case, showing openness to try new or different procedures or techniques, and assessing trainees’ baseline level of knowledge before giving new knowledge or explaining things. In the third part, the medical students were asked use a 5-point Likert scale (1 = strongly disagree, 5 = strongly agree) to express their level of satisfaction with the following: (1) receiving adequate training in ethics relevant to clinical decision in anesthesia, (2) improving their overall training or education experience, (3) participation in adequate collaborative research activities, (4) receiving hands-on training on how to use anesthesia equipment, (5) receiving adequate training that can enable them to communicate effectively and demonstrate caring and respectful behaviors when interacting with patients and their families, (6) receiving adequate training that can enable them to gather essential and accurate information about the patients, (7) receiving adequate training that can enable them to make informed decisions about anesthesia interventions based on clinical data, preferences of the patients, up-to-date scientific evidence, and clinical judgment, (8) receiving adequate training that can enable them to develop and carry out patient anesthesia management plans, (9) receiving adequate training that can enable them to counsel and educate patients and their families about anesthesia decisions, (10) receiving adequate training that can enable them to perform competently all medical and invasive procedures considered essential in the area of practice of anesthesia, 11) receiving adequate training that can enable them to provide healthcare services aimed at preventing health problems or maintaining health of patients in the practice of anesthesia, and 12) receiving adequate training that can enable them to work with healthcare professionals, including those from other disciplines, to provide patient-focused care.

### Validity and reliability of the study tool

The items included in the questionnaire were reviewed by a panel of anesthesiologists and academicians (*n = 6*) for face validity. Each item was rated for relevance and suitability using a Likert scale of 1–5 (1 = completely irrelevant/unsuitable, 5 = completely relevant/very suitable). The items that were rated completely relevant/relevant or very suitable were included in the final questionnaire. Discrepancies were resolved by discussion and consensus. A pilot test was conducted among 20 medical students to ensure the readability, clarity, and comprehensibility of the questionnaire. In the pilot test, the medical students were asked to fill out the questionnaire twice. The time interval between each administration was approximately 24 h. The internal consistency of the items was tested using Cronbach’s alpha statistics. It was decided *a priori* that a Cronbach’s alpha of ≥ 0.70 would indicate that the questionnaire was internally consistent. The stability of the answers of the participants was ensured using the test-retest method. Scores obtained in both rounds were correlated using Pearson’s correlations. It was decided *a priori* that a Pearson’s correlation coefficient (r) of ≥ 0.80 would indicate that the answers of the participants were stable.

### Statistical analysis

The data collected in this study were entered into IBM SPSS for Windows version 21.0. The data were assessed for normality of distribution using skewness and kurtosis values. As in previous studies, the data were considered normally distributed when the absolute kurtosis values were between − 7 and + 7 and the absolute skewness values were between − 2 and + 2 [18]. As the data were normally distributed, the central tendency was expressed using mean ± standard deviation (SD). Scores were compared using Student’s t-tests or analysis of variance (ANOVA), as appropriate. To control potentially confounding factors, a multiple linear regression was used. The goodness-of-fit was indicated by a statistically significant R^2^ (p-value < 0.05). Multicollinearity issues were diagnosed using variance inflation factor (VIF) and tolerance. In this study, a p-value of < 0.05 indicated statistical significance.

## Results

### Demographic, academic, and training variables of the medical students

In this study, questionnaires were returned by 385 medical students. The detailed demographic, academic, and training variables of the students are shown in Table 1. Of those, 223 (57.9%) were female and 186 (48.3%) were in their final year of medical training. The mean anesthesia rotation length was 12.4 ± 2.1 days and the mean surgery rotation length was 77.5 ± 17.1 days. On average, the students witnessed 7.8 ± 4.9 procedures performed under general, regional, and local anesthesia in a week.

**Table 1:** Demographic, academic, and training variables of the students (*n = 385*)

Variable	
Gender	
Male, n (%)	162 (42.1%)
Female n (%)	223 (57.9%)
**Age (years), Mean (± SD)**	23.2 (± 1.0)
**Academic year**	
5th, n (%)	199 (51.7%)
6th, n (%)	186 (48.3%)
**Length of anesthesia rotation (days), Mean (± SD)**	12.4 (± 2.1)
**Length of surgery rotation (days), Mean (± SD)**	77.5 (± 17.1)
**Number of students in anesthesia rotation, Mean (± SD)**	6.5 (± 2.5)
**Number of procedures performed under anesthesia (general, regional, local) in a week, Mean (± SD)**	7.8 (± 4.9)

Of the medical students, 92 (23.9%) were highly satisfied with their academic achievements, 281 (73.0%) self-rated their knowledge of anesthesia compared to other clinical subjects as adequate or higher, 46 (11.9%) of the medical students stated that they would consider a future career in anesthesia, and 142 (36.9%) stated that the rotation had a positive influence on their awareness of anesthesia. These results are shown in Table 2.

**Table 2:** Self-rated satisfaction, awareness, and knowledge about anesthesia

Statement	n (%)
Self-rated satisfaction with academic achievements	
Not satisfied, n (%)	32 (8.3%)
Neutral, n (%)	261 (67.8%)
Highly satisfied, n (%)	92 (23.9%)
**Were you aware of anesthesia as a specialty before your rotation?**	
No, n (%)	59 (15.3%)
Yes, n (%)	326 (84.7%)
**Self-rated knowledge of anesthesia compared to other clinical subjects**	
No knowledge, n (%)	12 (3.1%)
Poor, n (%)	92 (23.9%)
Adequate, n (%)	192 (49.9%)
Good, n (%)	80 (20.8%)
Excellent, n (%)	9 (2.3%)
**Would consider a future career in anesthesia**	
No, n (%)	89 (23.1%)
May be, n (%)	250 (64.9%)
Yes, n (%)	46 (11.9%)
**Self-rated influence of the anesthesia rotation on awareness of anesthesia**	
Negative influence, n (%)	29 (7.5%)
Neutral, n (%)	214 (55.6%)
Positive influence, n (%)	142 (36.9%)

### Teaching or training methods used by the educators during the anesthesia rotations

Of the students, 135 (35.1%) and 126 (32.8%) stated that the educators always or often explained how and why they did procedures or techniques during the anesthesia rotation and assessed their baseline level of knowledge before giving new knowledge or explaining things. On the other hand, stepping back and allowing the trainees to work through, presenting articles or literature relevant to the case, and being open to trying new or different procedures or techniques were less often reported by the medical students. Detailed views and opinions of the students on the teaching or training methods used during their anesthesia rotations are shown in Table 3.

**Table 3:** Views and opinions of the students on the teaching or training method

		Never		Rarely		Sometimes		Very often		Always	
#	Statement	n	%	n	%	n	%	n	%	n	%
1	During the anesthesia rotation, educators stepped back and allowed me to work through (direct patient care/gaining hands-on/preceptorship/mentorship)	23	6.0	76	19.7	193	50.1	68	17.7	25	6.5
2	During the anesthesia rotation, educators explained how and why they did procedures or techniques (case-based learning/problem-based learning/ debriefing session)	4	1.0	71	18.4	175	45.5	110	28.6	25	6.5
3	During the anesthesia rotation, educators presented articles or literature relevant to the case (journal clubs/small group discussions)	41	10.6	97	25.2	159	41.3	64	16.6	24	6.2
4	During the anesthesia rotation, educators were open to trying new or different procedures or techniques (case-based learning/bedside teaching)	19	4.9	101	26.2	166	43.1	74	19.2	25	6.5
5	During the anesthesia rotation, educators assessed my baseline level of knowledge before giving new knowledge or explaining things (objective assessments/procedural checklists/rounds and case presentations)	19	4.9	79	20.5	161	41.8	90	23.4	36	9.4

### Satisfaction of the medical students with the anesthesia rotation

In this study, less than half of the medical students agreed or strongly agreed that they have received adequate training that can enable them to communicate effectively, demonstrate caring, and respectful behaviors, gather essential and accurate information, make informed decisions about anesthesia interventions, work with other healthcare professionals, provide patient-focused care, provide healthcare services aimed at preventing health problems, perform competently all essential anesthesia procedures, develop and carry out patient anesthesia management plans, and counsel or educate patients about anesthesia decisions. Similarly, a minority of the medical students agreed or strongly agreed that they have received hands-on training on how to use anesthesia equipment, the rotation has improved their overall training or education experience, have received adequate training in ethics relevant to the clinical decision in anesthesia, and have participated in adequate collaborative research activities. Detailed answers of the medical students are shown in Table 4.

**Table 4:** Views and opinions of the medical students regarding the anesthesia rotation

		Strongly disagree		Disagree		Neutral		Agree		Strongly agree	
#	Statement	n	%	n	%	n	%	n	%	n	%
1	After completing my anesthesia rotation, I feel that I have received adequate training in ethics relevant to the clinical decision in anesthesia	36	9.4	67	17.4	180	46.8	73	19.0	29	7.5
2	After completing my anesthesia rotation, I feel that this rotation has improved my overall training and education experience	21	5.5	77	20.0	163	42.3	110	28.6	14	3.6
3	After completing my anesthesia rotation, I feel that I have participated in adequate collaborative research activities	59	15.3	116	30.1	122	31.7	73	19.0	15	3.9
4	After completing my anesthesia rotation, I feel that I have received hands-on training on how to use anesthesia equipment	25	6.5	89	23.1	124	32.2	108	28.1	39	10.1
5	After completing my anesthesia rotation, I feel that I have received adequate training that can enable me to communicate effectively and demonstrate caring and respectful behaviors when interacting with patients and their families	23	6.0	49	12.7	134	34.8	151	39.2	28	7.3
6	After completing my anesthesia rotation, I feel that I have received adequate training that can enable me to gather essential and accurate information about the patients	7	1.8	67	17.4	137	35.6	153	39.7	21	5.5
7	After completing my anesthesia rotation, I feel that I have received adequate training that can enable me to make informed decisions about anesthesia interventions based on clinical data, preferences of the patients, up-to-date scientific evidence, and clinical judgment	14	3.6	72	18.7	136	35.3	136	35.3	27	7.0
8	After completing my anesthesia rotation, I feel that I have received adequate training that can enable me to develop and carry out patient anesthesia management plans	16	4.2	75	19.5	154	40.0	118	30.6	22	5.7
9	After completing my anesthesia rotation, I feel that I have received adequate training that can enable me to counsel and educate patients and their families about anesthesia decisions	15	3.9	75	19.5	146	37.9	125	32.5	24	6.2
10	After completing my anesthesia rotation, I feel that I have received adequate training that enable me to perform competently all medical and invasive procedures considered essential in the area of practice of anesthesia^*^	17	4.4	90	23.4	139	36.1	117	30.4	22	5.7
11	After completing my anesthesia rotation, I feel that I have received adequate training that can enable me to provide healthcare services aimed at preventing health problems or maintaining the health of patients in the practice of anesthesia	15	3.9	89	23.1	134	34.8	126	32.7	21	5.5
12	After completing my anesthesia rotation, I feel that I have received adequate training that can enable me to work with healthcare professionals, including those from other disciplines, to provide patient-focused care	14	3.6	77	20.0	142	36.9	137	35.6	15	3.9

^*^The rotation is expected to achieve the intended learning outcomes including pre-operative assessment of patients, airway and fluid management, types of anesthesia, indications, and contraindications of anesthesia, induction and maintenance of anesthesia, complications associated with anesthesia, monitoring and recovery of patients, differential diagnosis of hypoxemia and hypercarbia, indication and practice of mechanical ventilation and resuscitation, pain management, preparation of a care plan for patients undergoing general and regional anesthesia, how to deal with difficult intubation procedures, and how to deal with high-risk patients

### Differences in satisfaction scores in relation to the demographic, academic, and training variables of the medical students

Student’s t-tests and ANOVA showed that the satisfaction scores were significantly higher for the medical students who were male, in their 6th year of medical training, expressed high satisfaction with their academic achievements, self-rated their knowledge of anesthesia as excellent compared to other clinical subjects, were willing to consider a future career in anesthesia, self-rated a positive influence of the anesthesia rotation on their awareness of anesthesia, and reported frequent active or adequate teaching or training methods by educators. Differences in satisfaction scores in relation to the demographic, academic, and training variables of the medical students are shown in Table 5.

**Table 5:** Differences in satisfaction scores in relation to the demographic, academic, and training variables of the medical students

Variable	n	%	Mean	SD	p-value
Gender					
Male	162	42.1	66.3	14.0	< 0.001^*^
Female	223	57.9	59.2	14.4	
**Academic year**					
5th year	199	51.7	59.3	14.1	< 0.001^*^
6th year	186	48.3	65.3	14.6	
**Self-rated satisfaction with academic achievements**					
Not satisfied	261	67.8	61.7	2.7	< 0.001^**^
Neutral	32	8.3	60.1	0.9	
Highly satisfied	92	23.9	68.6	1.3	
**Self-rated knowledge of anesthesia compared to other clinical subjects**					
No knowledge	92	23.9	60.3	4.6	< 0.001^**^
Poor	12	3.1	59.1	1.6	
Adequate	192	49.9	60.6	1.0	
Good	9	2.3	69.3	1.5	
Excellent	80	20.8	68.1	5.4	
**Willingness to consider a future career in anesthesia**					
No	89	23.1	61.0	1.5	0.002^**^
May be	250	64.9	61.4	0.9	
Yes	46	11.9	69.2	2.1	
**Self-rated influence of the anesthesia rotation on awareness of anesthesia**					
Negatively	29	7.5	60.6	3.4	< 0.001^**^
Neutral	214	55.6	58.9	1.0	
Positively	142	36.9	67.5	1.1	
**Frequency of educators stepping back and allowing the trainee to work through**					
Never	23	6.0	49.1	3.6	< 0.001^**^
Rarely	76	19.7	52.9	1.6	
Sometimes	193	50.1	61.4	0.8	
Very often	68	17.7	73.7	1.3	
Always	25	6.5	77.7	2.3	
**Frequency of explaining how and why the educators did procedures or techniques**					
Never	4	1.0	39.2	5.4	< 0.001^**^
Rarely	71	18.4	48.8	1.5	
Sometimes	175	45.5	60.7	0.9	
Very often	110	28.6	71.5	1.1	
Always	25	6.5	74.0	2.1	
**Frequency of presenting articles or literature relevant to the case by the educators**					
Never	41	10.6	53.0	2.3	< 0.001^**^
Rarely	97	25.2	55.9	1.4	
Sometimes	159	41.3	61.8	0.9	
Very often	64	16.6	73.4	1.6	
Always	24	6.2	77.0	2.1	
**Frequency of openness of educators to try new or different procedures or techniques**					
Never	19	4.9	49.1	4.1	< 0.001^**^
Rarely	101	26.2	54.1	1.3	
Sometimes	166	43.1	61.7	0.9	
Very often	74	19.2	73.1	1.3	
Always	25	6.5	76.4	2.6	
**Frequency of assessing the baseline level of knowledge of the trainees before giving new knowledge or explaining things by the educators**					
Never	19	4.9	43.5	3.3	< 0.001^**^
Rarely	79	20.5	52	1.2	
Sometimes	161	41.8	61.2	1.0	
Very often	90	23.4	72.1	1.2	
Always	36	9.4	74.4	2.2	

^*^Student’s t-test, ^**^ANOVA

Multiple linear regression showed that higher satisfaction scores were predicted by self-rating knowledge of anesthesia as excellent compared to other clinical subjects, reporting frequent educators stepping back and allowing the trainee to work through, explaining how and why the educators did procedures or techniques, presenting articles or literature relevant to the case by the educators, and assessing baseline level of knowledge of the trainees before giving new knowledge or explaining things by the educators. Details of the multiple regression model are shown in Table 6.

**Table 6:** Predictors of satisfaction scores^*^

Variable	Unstandardized Coefficients	SE	Standardized Coefficients	t	p-value
Gender	-1.81	1.27	-0.06	-1.42	0.156
Age	-1.25	0.70	-0.09	-1.78	0.076
Academic year	-1.55	1.61	-0.05	-0.96	0.338
Length of anesthesia rotation	0.46	0.40	0.07	1.16	0.249
Length of surgery rotation	0.05	0.05	0.05	0.99	0.324
Self-rated satisfaction with academic achievements	0.68	0.62	0.04	1.09	0.275
Self-rated knowledge of anesthesia compared to other clinical subjects	2.17	0.74	0.12	2.92	0.004
Willingness to consider a future career in anesthesia	-1.30	1.07	-0.05	-1.21	0.227
Self-rated influence of the anesthesia rotation on awareness of anesthesia	1.35	1.00	0.06	1.35	0.178
Number of procedures performed under anesthesia	0.24	0.14	0.08	1.67	0.096
Frequency of educators stepping back and allowing the trainee to work through	1.74	0.80	0.11	2.18	0.030
Frequency of explaining how and why the educators did procedures or techniques	4.33	0.87	0.25	4.98	< 0.001
Frequency of presenting articles or literature relevant to the case by the educators	2.04	0.79	0.14	2.58	0.010
Frequency of openness of educators to try new or different procedures or techniques	1.30	0.90	0.08	1.44	0.150
Frequency of assessing the baseline level of knowledge of the trainees before giving new knowledge or explaining things by the educators	2.66	0.80	0.18	3.32	0.001

^*^Multiple linear regression

## Discussion

In clinical practice, anesthesiologists provide preoperative, intraoperative, and postoperative healthcare services to patients. To be able to proficiently perform practical procedures, anesthesiologists should be trained adequately. This study was conducted to evaluate anesthesia rotations and assess the satisfaction of the trainees in Palestine. To the best of our knowledge, this was the first study to determine the teaching and training approaches used by the trainers during the anesthesia rotations and the satisfaction of the trainees. The findings reported in this study are informative to educators, anesthesiologists, professional bodies, and decision-makers in academia and healthcare authorities who could be interested in improving the education and training of medical students in anesthesia.

In this study, the anesthesia trainees reported that the majority of the educators did not assess the baseline level of knowledge of the trainees before giving new knowledge or explaining things. Slater et al. proposed a framework for an ideal learning scenario [19]. The framework was proposed to be used in preclinical and clinical environments. Probably, the educators might need to consider the trainee as a novice and attempt to engage them through multiple means of representations, actions, and expressions [20]. Formative assessment and probing questions that could be followed by explaining how and why the educators did procedures or techniques could facilitate learning and gaining new knowledge. Additionally, the anesthesia trainees reported that the educators used effective teaching or training approaches less often than expected during anesthesia rotations. Previous studies have shown that preceptorships/mentorships, simulation-based training, case-based learning, bedside teaching, evidence-based learning, and conducting debriefing sessions could improve the learning outcomes in anesthesia [21–23]. The majority of the anesthesia trainees (77.1%) reported that the educators did not often present articles or literature relevant to the case. Research articles, reviews, and case reports are valuable tools in clinical learning environments. Presenting relevant articles, reviews, and case reports would help provide an enriching learning experience [24]. Similarly, the majority of the anesthesia trainees (75.8%) reported that the educators did not often step back and allow the trainees to work through. Many procedures in anesthesiology are considered “open skills” in that they require anesthesiologists to adapt to unpredictable and ever-changing conditions [25]. Although not all students would be expected to pursue a career in anesthesia, exposure to different practices, specificity of learning, receiving or observing immediate feedback, awareness of outcomes, and observational practice could improve the learning/training experiences of medical students [25, 26]. Although the safety of the patients would prevail over the training of students, educators are encouraged to use every opportunity to step back and allow the trainees to work through while safeguarding the safety of the patients. In this study, the majority of the anesthesia trainees (74.2%) reported that the educators were not open to trying new or different procedures or techniques. Being open to trying new or different procedures or techniques is encompassed by adaptation to unpredictable and ever-changing conditions in clinical practice [25]. This can also help trainees gain hands-on skills in these new procedures and techniques.

Communicating effectively with the patients and families, demonstrating caring and respectful behaviors to the patients and their families, gathering essential and accurate information, making informed decisions about anesthesia interventions, working with other healthcare professionals, providing patient-focused care, providing healthcare services, performing competently all essential anesthesia procedures, developing and carrying out patient anesthesia management plans, and counseling or educating patients about anesthesia decisions are essential competencies in anesthesiology [1–4, 12–15]. In this study, less than half of the anesthesia trainees expressed satisfaction with these competencies after their anesthesia rotation. In this study, male students expressed higher satisfaction than their female counterparts. Probably, male students are more likely to engage in discussions and hands-on gaining activities compared to female students in the Palestinian context. These findings indicate that educators should pay more attention to engaging female students in different learning activities. Additionally, the students who were in their 6th year of medical training were satisfied with their academic achievements, rated their knowledge of anesthesia as excellent, expressed willingness to consider a future career in anesthesia, perceived a positive influence of the anesthesia rotation, and reported frequent active teaching or training methods by their educators expressed higher satisfaction. These findings were not surprising as advancing in medical education, higher academic achievements, and interest in anesthesia as a future career could have improved the experiences of those students during the anesthesia rotation.

The findings of this study indicate that educators and other decision-makers should consider developing a more satisfying experience for the medical students who attend anesthesia rotations. Probably, using more engaging and active learning techniques might improve the satisfaction of the trainees [2, 3, 8, 13, 25]. These engaging and active learning techniques should focus on providing opportunities for the trainees to gain hands-on skills in using anesthesia equipment and engage in collaborative research activities. Additionally, the trainees should receive training in ethics relevant to the clinical decision in anesthesia. Probably, more efforts should be paid to improve the satisfaction of the trainees who rated their knowledge of anesthesia as less than excellent and whose educators did not use effective and engaging teaching or training techniques.

## Strengths and limitations of the study

The findings reported in this study might be interpreted after considering some strengths and limitations. First, this is the first study to evaluate teaching or training approaches used in anesthesia rotations and the satisfaction of the trainees. Second, a large sample size was recruited in this study. Additionally, the participants were diversified in terms of gender, length of anesthesia rotation, satisfaction with academic achievement, and knowledge of anesthesia. This diversity should have ensured the representativeness of the entire population of medical students and the external validity of the findings. Third, the participants were recruited from different universities offering medical education in Palestine. Again, this should have ensured the representativeness of the entire population of medical students and the external validity of the findings. Fourth, the study tool was tested for reliability and internal consistency in a pilot study before it was used.

This study is not without limitations. First, the study was conducted in a cross-sectional design. The findings of cross-sectional studies are limited by the period in which they were conducted. Second, recall bias could not be excluded from this study as some 6th year students have completed their anesthesia rotation one year before this study was conducted. Third, desirability bias could not be excluded as some students might have tended to provide more positive answers.

## Conclusions

Educators used active and effective teaching or training approaches less frequently during anesthesia rotations. The findings of this study also showed that the medical students were not satisfied with their competencies after their anesthesia rotations. The findings of this study might be informative to educators, decision, and policymakers in academia and medical professional groups who might need to plan and implement strategies to improve anesthesia rotations and medical education or training. More studies are still needed to determine the best ways to improve anesthesia rotations and medical education or training.

## Data Availability

All data analyzed in this study were included in the manuscript.

## References

[1] Starkie T, Drake EJ (2013). Assessment of procedural skills training and performance in anesthesia using cumulative sum analysis (cusum). Can J Anaesthesia = Journal Canadien D’anesthesie.

[2] Oliveira KF, Arzola C, Ye XY, Clivatti J, Siddiqui N, You-Ten KE (2017). Determining the amount of training needed for competency of anesthesia trainees in ultrasonographic identification of the cricothyroid membrane. BMC Anesthesiol.

[3] Dobson G, Chow L, Filteau L, Flexman A, Hurdle H, Kurrek M, Milkovich R, Perrault MA, Sparrow K, Swart PA (2020). Guidelines to the practice of anesthesia - revised Edition 2020. Can J Anaesthesia = Journal Canadien D’anesthesie.

[4] Greiner A, Knebel E (2003). The core competencies needed for health care professionals. Health professions education: a bridge to quality.

[5] Al Ghofaily L, Mitchell JD, Woodworth G (2018). Anesthesia residency training in Cardiac Anesthesia: development of a Model Curricula and Educational Resources: the Anesthesia Toolbox. J Cardiothorac Vasc Anesth.

[6] Van Melle E, Hall AK, Schumacher DJ, Kinnear B, Gruppen L, Thoma B, Caretta-Weyer H, Cooke LJ, Frank JR (2021). Capturing outcomes of competency-based medical education: the call and the challenge. Med Teach.

[7] Tetzlaff JE, Warltier DC (2007). Assessment of competency in anesthesiology. J Am Soc Anesthesiologists.

[8] Delphin E, Davidson M (2008). Teaching and evaluating group competency in systems-based practice in anesthesiology. Anesth Analg.

[9] Hausman LM, Levine AI, Rosenblatt MA (2006). A survey evaluating the training of anesthesiology residents in office-based anesthesia. J Clin Anesth.

[10] Shawahna R, Jaber M, Yahya N, Jawadeh F, Rawajbeh S (2021). Are medical students in Palestine adequately trained to care for individuals with autism spectrum disorders? A multicenter cross-sectional study of their familiarity, knowledge, confidence, and willingness to learn. BMC Med Educ.

[11] Vandenbroucke JP, von Elm E, Altman DG, Gøtzsche PC, Mulrow CD, Pocock SJ, Poole C, Schlesselman JJ, Egger M (2007). Strengthening the reporting of Observational Studies in Epidemiology (STROBE): explanation and elaboration. PLoS Med.

[12] Elobu AE, Kintu A, Galukande M, Kaggwa S, Mijjumbi C, Tindimwebwa J, Roche A, Dubowitz G, Ozgediz D, Lipnick M (2014). Evaluating international global health collaborations: perspectives from surgery and anesthesia trainees in Uganda. Surgery.

[13] Wakatsuki S, Tanaka P, Vinagre R, Marty A, Thomsen J, Macario A (2018). What makes for good anesthesia teaching by Faculty in the operating room? The perspective of Anesthesiology residents. Cureus.

[14] Tetzlaff JE (2007). Assessment of competency in anesthesiology. Anesthesiology.

[15] Kopp SL, Smith HM (2011). Developing effective web-based regional anesthesia education: a randomized study evaluating case-based versus non-case-based module design. Reg Anesth Pain Med.

[16] Kibwana S, Woldemariam D, Misganaw A, Teshome M, Akalu L, Kols A, Kim YM, Mengistu S, van Roosmalen J, Stekelenburg J. Preparing the Health workforce in Ethiopia: a cross-sectional study of competence of Anesthesia graduating students. Educ Health 2016, 29(1). 10.4103/1357-6283.178931 26996792

[17] Spijkerman S, Manning DM, Green-Thompson LP. Undergraduate Anesthesia Skills for a Global Surgery Agenda: Students’ Self-Reported Competence. Anesth Analg; 2023. 10.1213/ANE.0000000000006375 36888537

[18] George D, Mallery P. IBM SPSS statistics 26 step by step: a simple guide and reference. Routledge; 2019.

[19] Slater RJ, Castanelli DJ, Barrington MJ (2014). Learning and teaching motor skills in regional anesthesia: a different perspective. Reg Anesth Pain Med.

[20] Kannan J, Kurup V (2012). Blended learning in anesthesia education: current state and future model. Curr Opin Anaesthesiol.

[21] Chilkoti G, Mohta M, Wadhwa R, Saxena AK. Problem-based Learning Research in Anesthesia Teaching: current status and future perspective. Anesthesiol Res Pract 2014, 2014:263948. 10.1155/2014/263948 PMC4058836 24982673

[22] Chilkoti G, Wadhwa R, Kumar A (2015). Status of problem based learning in postgraduate anesthesia teaching: a cross-sectional survey. Saudi J Anaesth.

[23] Nelsen BR, Chen Y-YK, Lasic M, Bader AM, Arriaga AF. Advances in anesthesia education: increasing access and collaboration in medical education, from E-learning to telesimulation. Curr Opin Anesthesiology 2020, 33(6). 10.1097/ACO.0000000000000931 33060385

[24] Kim TE, Tsui BCH (2019). Simulation-based ultrasound-guided regional anesthesia curriculum for anesthesiology residents. Korean J Anesthesiology.

[25] Cordovani L, Cordovani D (2016). A literature review on observational learning for medical motor skills and anesthesia teaching. Adv Health Sci Education: Theory Pract.

[26] Wulf G, Shea C, Lewthwaite R (2010). Motor skill learning and performance: a review of influential factors. Med Educ.

